# CD209d/e promotes inflammation and lung injury during influenza virus infection

**DOI:** 10.1093/immhor/vlae001

**Published:** 2025-01-23

**Authors:** Radha Gopal, Michael A Marinelli, Flavia Rago, Lacee J Richwalls, Nicholas J Constantinesco, Deepa Debnath, Saran Kupul, Maria de la Luz Garcia-Hernandez, Javier Rangel-Moreno, Jay K Kolls, John F Alcorn

**Affiliations:** Division of Pulmonary Medicine, Department of Pediatrics, UPMC Children’s Hospital of Pittsburgh, Pittsburgh, PA, United States; Division of Pulmonary Medicine, Department of Pediatrics, UPMC Children’s Hospital of Pittsburgh, Pittsburgh, PA, United States; Division of Pulmonary Medicine, Department of Pediatrics, UPMC Children’s Hospital of Pittsburgh, Pittsburgh, PA, United States; Division of Pulmonary Medicine, Department of Pediatrics, UPMC Children’s Hospital of Pittsburgh, Pittsburgh, PA, United States; Division of Pulmonary Medicine, Department of Pediatrics, UPMC Children’s Hospital of Pittsburgh, Pittsburgh, PA, United States; Division of Pulmonary Medicine, Department of Pediatrics, UPMC Children’s Hospital of Pittsburgh, Pittsburgh, PA, United States; Division of Pulmonary Medicine, Department of Pediatrics, UPMC Children’s Hospital of Pittsburgh, Pittsburgh, PA, United States; Division of Allergy, Immunology and Rheumatology, Department of Medicine, University of Rochester, Rochester, NY, United States; Division of Allergy, Immunology and Rheumatology, Department of Medicine, University of Rochester, Rochester, NY, United States; Center for Translational Research in Infection and Inflammation, Tulane University School of Medicine, New Orleans, LA, United States; Division of Pulmonary Medicine, Department of Pediatrics, UPMC Children’s Hospital of Pittsburgh, Pittsburgh, PA, United States; Department of Immunology, University of Pittsburgh, Pittsburgh, PA, United States

**Keywords:** DC-SIGN, mice, pattern recognition receptor, pneumonia, virus

## Abstract

Influenza virus infects millions each year, contributing greatly to human morbidity and mortality. Upon viral infection, pathogen-associated molecular patterns activate pattern recognition receptors on host cells, triggering an immune response. The CD209 protein family, homologs of DC-SIGN (dendritic cell–specific intercellular adhesion molecule 3–grabbing nonintegrin), is thought to modulate immune responses to viruses. The effects of the mouse functional DC-SIGN homolog CD209d/e on the lung immune responses during influenza viral infection are not known. Therefore, we generated mice that lack both CD209d and e isoforms to determine the role in influenza viral infection. We infected wild-type and CD209d/e gene–deficient (CD209d/e^−/−^) mice with influenza virus and measured the cellular response in bronchoalveolar lavage, the expression of proinflammatory cytokines, antiviral genes, toll-like receptors (TLRs) in the lung, and lung pathology. We found CD209d/e^−/−^ mice had decreased viral burden, TLR3 and TLR9 expression, interferon response, macrophages in bronchoalveolar lavage, and parenchymal lung inflammation compared with control mice. We also found less influenza viral uptake in alveolar macrophages and bone marrow–derived macrophages isolated from CD209d/e^−/−^ mice when compared with control mice. We further investigated the role CD209d/e by treating bone marrow–derived macrophages from control and CD209d/e^−/−^ mice with TLR agonists. We found that lacking CD209d/e decreased the expression of TLR3, TLR9, RIG1, STAT1, and STAT2 compared with controls. Collectively these results show that CD209d/e plays an important role in viral sensing/uptake and inflammatory immune responses during influenza viral infection.

## Introduction

Influenza virus infection contributes significantly to worldwide respiratory morbidity, mortality, and costs relating to treatment and lost earnings. Each year, millions of people become infected with influenza virus, which results in millions of outpatient visits, hundreds of thousands of hospitalizations, and tens of thousands of deaths in the United States alone. Influenza-associated costs for treatment and lost workdays are estimated to be tens of billions of dollars annually, during nonpandemic years.^1^ During the most recent pandemic in 2009, it is estimated that up to 24% of the world, over 1.6 billion people, became infected with the virus.^2^ Despite its low mortality rate; influenza remains a top 10 cause of death in Americans 1 to 24 and 65 years of age and older.^3^ Further, secondary bacterial infections, with pathogens such as methicillin-resistant *Staphylococcus aureus*, associated with influenza contribute to an increase in rates of mortality and morbidity.^4^

Influenza virus is sensed by host cells through pattern recognition receptors. Toll-like receptors (TLRs); retinoic acid inducible gene I-like receptors (RLRs); nucleotide-binding domain, leucine-rich containing receptors (NLRs), and C-type lectin receptors are the major pattern recognition receptors involved in sensing pathogen-associated molecular patterns, thereby activating downstream signaling pathways and inducing inflammatory cytokines and interferons.^5^^,^^6^ Dendritic cell–specific intercellular adhesion molecule 3–grabbing nonintegrin (DC-SIGN), also known as cluster of differentiation 209 (CD209) is a C-type lectin membrane protein found commonly in the cell membrane of dendritic cells and macrophages of the immune system. It can identify microbes, including bacteria and viruses, by binding to mannose oligosaccharides and fucose-containing structures of the pathogen. In humans, the CD209 family has 2 members, dendritic cell CD209 (DC-SIGN) and liver/lymph node CD209L (L-SIGN) that differ based on the cell type they are found on and in function. Mice have 8 homologs of these proteins (specific intracellular adhesion molecule 3–grabbing nonintegrin receptors 1–8 [SIGNR]).^7^ It is believed that murine SIGNR3 (CD209d) and human CD209 are the original members of this receptor family that have undergone different evolutionary paths. The SIGNR3 gene in mouse has become duplicated resulting in more homologs with diverse functions in innate recognition.^8^^,^^9^ Herein, we have focused on CD209d because it is the most similar murine homolog of human DC-SIGN in terms of ligand recognition and function.^9^ Additionally, CD209d/e were among the most differentially regulated genes in STAT2^−/−^ mice infected with influenza virus and methicillin-resistant *S. aureus* superinfection, suggesting that they are responsive to viral induced interferon signaling.^10^ This study examined the role of SIGNR3 (CD209d) and SIGNR4 (CD209e) in the immune response to influenza viral infection and its relation to viral burden and lung inflammation, in wild-type (WT) and CD209d/e gene–deficient (CD209d/e^−/−^) phagocytes and mice.

## Materials and methods

### Mice

WT C57BL/6 (6–8 wk old) mice were purchased from Taconic Farms. CD209d/e^*−/−*^ mice on C57BL/6 background were generated from the Innovative Technologies Development Core at the University of Pittsburgh, and colonies were subsequently maintained under specific pathogen–free conditions. In vivo studies were performed on age matched adult male mice, unless otherwise indicated. All experiments were approved by the University of Pittsburgh Institutional Animal Care and Use Committee.

### Generation of CD209d/e^−/−^ mice and validation

CD209d/e^*−/−*^ mice on C57BL/6 background were generated from zygotes from a C57BL/6J donor (Jackson Laboratory). A total of 237 C57BL/6J zygotes were micro injected with a mixture of 100 ng/µL Cas9 mRNA, CD209d sgRNA 50 ng/µL, and Cd209e sgRNA 50 ng/µL. From the injected zygotes, 168 embryos developed to the 2-cell stage and were transferred to the oviducts of 6 CD1 (Charles River Laboratories) pseudopregnant female recipients. Thirteen live births occurred from those transfers. Potential founder mice were toe clipped for identification by genotyping. The potential founder mice were genotyped with the primer pair CD209d/e^−/−^ F52, 5′AGCTGTGTGACTATGAGCCTG3′, and CD209d/e^−/−^ R32, 5′-GGGACTTTCCAATGG GCTCT3′ to detect mice bearing a deleted allele. Mice 1, 4, and 5 were shown to carry a deletion fragment of approximately 430 bp. Once the mice were genotyped, the polymerase chain reaction (PCR) products were purified with ExoSAP-IT for PCR Product Cleanup (Affymetrix) and sequenced with forward and reverse primers, CD209d/e^−/−^ F52 and CD209d/e^−/−^ R32, to confirm PCR product specificity and determine deletion border junctions. Forward and reverse sequences for each mouse were aligned and the contigs were aligned against the mouse genomic reference sequence (UCSC Genome Browser Mouse Dec. 2011 [GRCm38/mm10] Assembly). Additional information is included in Material S1.

### Influenza a PR/8/34 H1N1 virus infection

Mice were infected with 100 plaque-forming units (PFUs) of Influenza A/PR/8/34 (H1N1) virus in 50 µL of sterile phosphate-buffered saline (PBS) from a frozen stock grown from chicken eggs.^10^^,^^11^ Infections were administered on isoflurane-anesthetized mice using oropharyngeal aspiration. Infected mice were incubated for 7 d. On the seventh day, mouse tissues were harvested and bronchoalveolar lavage (BAL) fluid, lungs, and serum were collected. Quantitative real-time reverse-transcriptase PCR (RT-PCR)was used to determine viral burden in lung RNA based on the amount of viral RNA (M protein) using forward primer: 5′-GGACTGCAGCGTAGACGCTT-3′; reverse primer: 5′-CATCCTGTTGTATATGAGGCCCAT-3′; probe: 5′-/56-FAM/CTCAGTTAT/ZEN/TCTG CTG GTGCACTTGCCA/3IABkF Q/−3′; and plaque assay.^12–14^

### Lung inflammation measurement

BAL fluid was collected by flushing the lungs of mice with 1 mL of sterile PBS. BAL cells were affixed to slides via cytospin and slides were stained with Protocol Hema 3 staining (Thermo Fisher Scientific) and used for differential cell counts. The middle lobes of the right lung were collected and snap frozen with liquid nitrogen. These were homogenized mechanically, and RNA was extracted using the Absolutely RNA Miniprep Kit (Agilent Technologies). Gene expression was measured using RT-PCR with TaqMan primer and probes (Thermo Fisher Scientific) (Table S1). Expression was calculated using the delta-delta CT method and was normalized to the housekeeping gene HPRT.^15^ The left lobe of the lung was pressure inflated and fixed in 10% neutral-buffered formalin for histology. Histological slides were blinded and scored according to the inflammatory cellular accumulation in lung parenchymal, peribronchial, and perivascular areas by a sample-blinded pathologist as described.^16–18^

### Influenza viral binding assay

**Figure 1.**
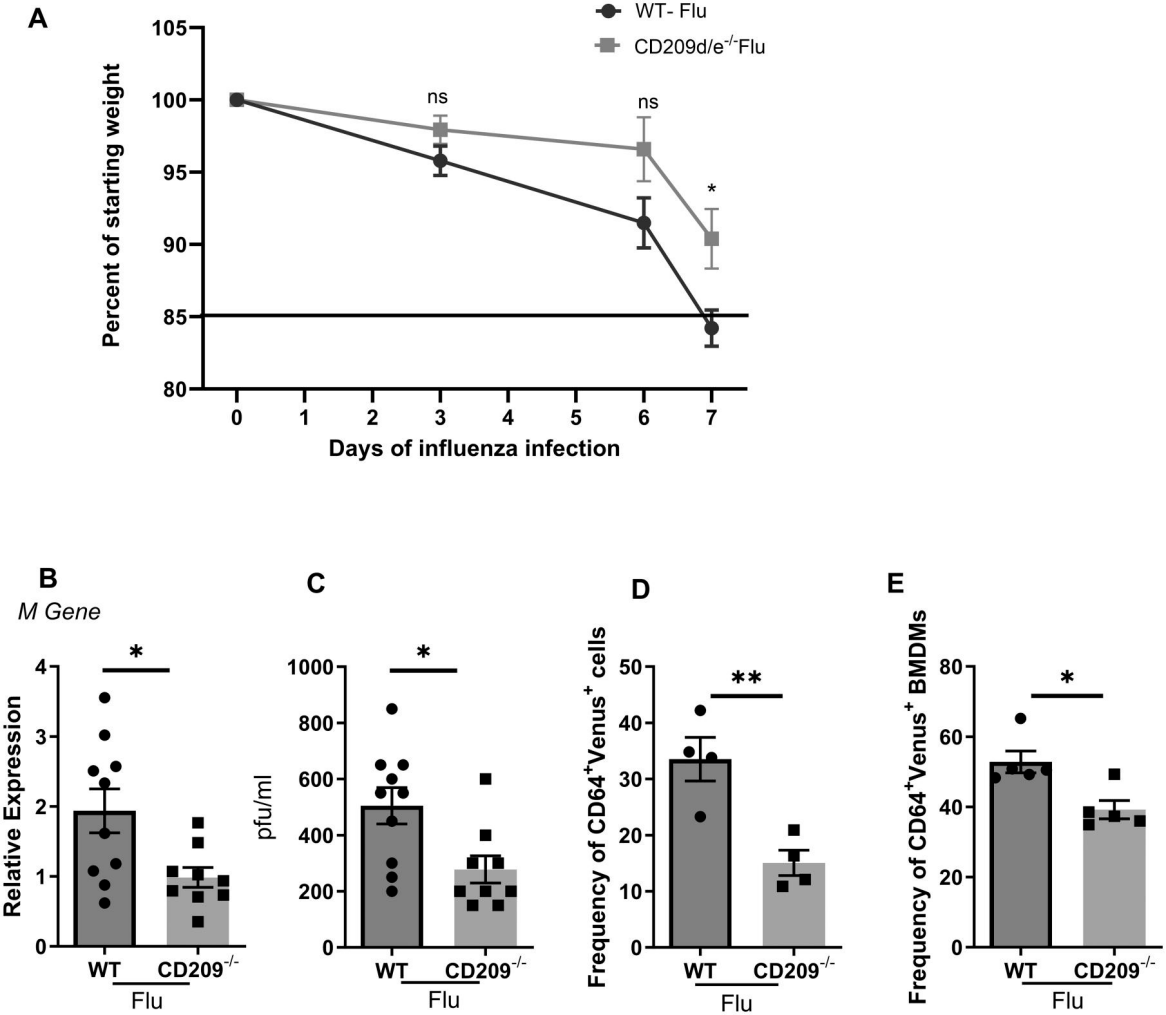
CD209d/e are required for influenza virus infection in the lung and in macrophages. WT and CD209d/e^−/−^ (CD209^−/−^) mice were infected with 10^2^ PFUs of influenza A PR/8/34 virus (Flu) (A) Weight loss was determined by comparing weight on days 3, 6, and 7 of infection. (B, C) Expression of influenza viral M protein and viral titer on day 7 postinfection was measured in the lung by RT-PCR and plaque assay, respectively. BAL cells were isolated from WT and CD209d/e^−/−^ mice and infected with Color-flu (Venus, multiplicity of infection of 1). (D) The frequency of CD64^+^ Venus^+^ cells were analyzed by flow cytometry. BMDMs were isolated from WT and CD209d/e^−/−^ mice and infected with Color-flu (Venus, multiplicity of infection of 1). (E) The frequency of CD64^+^ Venus^+^ cells were analyzed by flow cytometry. Data are represented as mean ± SEM. Significance was tested by unpaired *t* test. Each experiment was independently performed 2 or more times, and combined data are shown. *P < 0.05, **P < 0.01.

**Figure 2.**
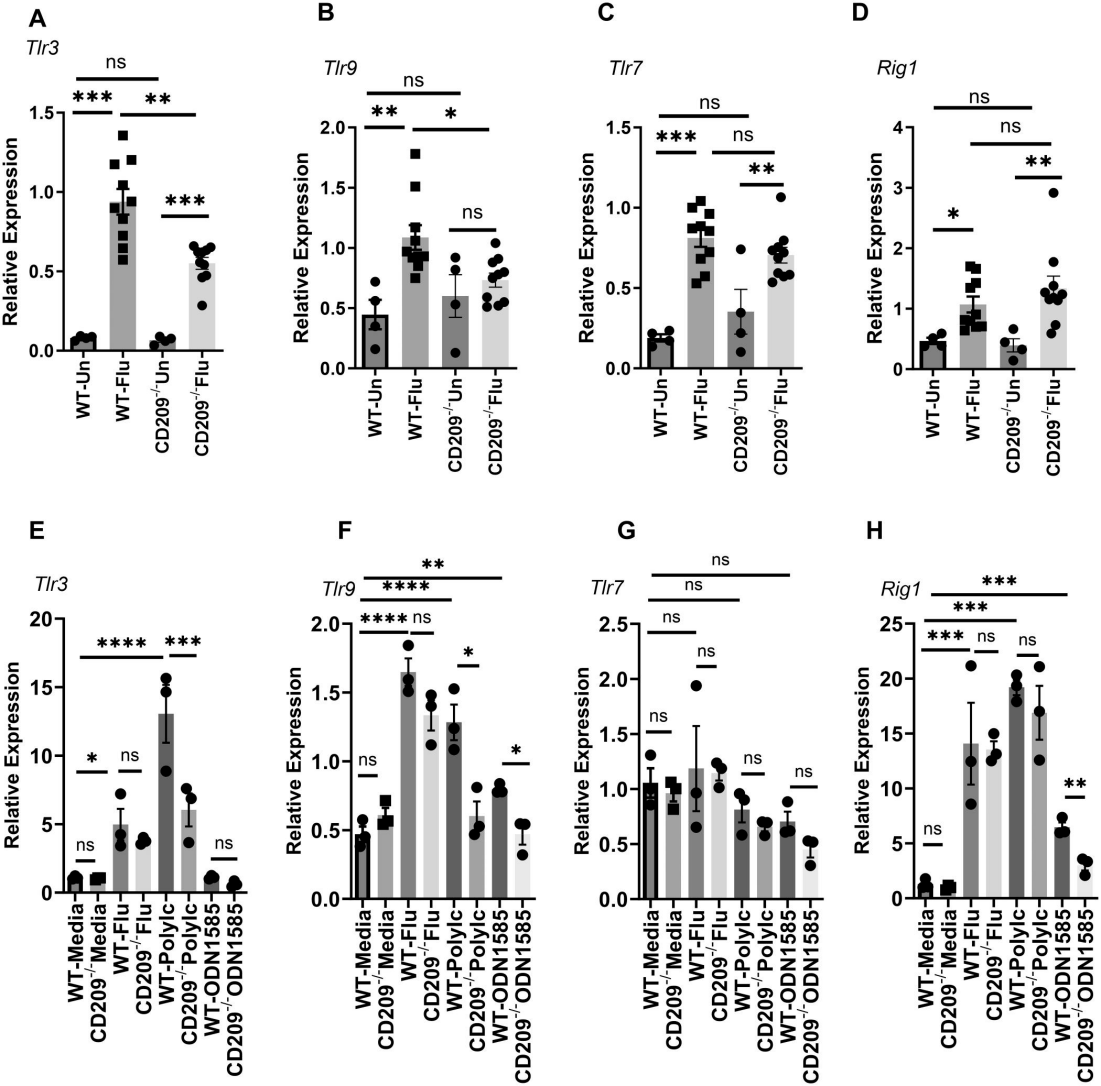
TLR expression is suppressed in CD209d/e−/− mice during influenza virus infection. WT and CD209d/e^−/−^ (CD209^−/−^) mice were infected with 10^2^ PFUs of influenza A PR/8/34 virus (Flu) for 7 d. (A–D) *Tlr3*, *Tlr9*, *Tlr7*, and *Rig1* expression levels in whole lung were measured from uninfected (Un) and influenza-infected mice by RT-PCR. (E–H) BMDMs from WT and CD209d/e^−/−^ mice were treated with influenza A/PR/8/34 virus, Poly I:C (10 µg/mL), or CpG ODN1585 (5 µM/mL) for 24 h, and *Tlr3*, *Tlr9*, *Tlr7*, and *Rig1* expression levels were measured by RT-PCR. Data are represented as mean ± SEM. Significance was tested by analysis of variance and Tukey test. Each experiment was independently performed 2 or more times, and combined data are shown. *P < 0.05, **P < 0.01, ***P < 0.001, ****P < 0.0001. ns, not significant.

BAL cells from naïve mice were isolated as described.^19^ The cells were washed, counted, and seeded on the cell culture plate overnight in 5% CO_2_ at 37°C. The cells (2 × 10^5^) were infected with Color-flu H1N1 (Venus, multiplicity of infection of 1) for 4 hours in serum-free media in 5% CO_2_ at 37°C. The Color-flu virus was a kind gift from Dr. Yoshihiro Kawaoka at the University of Wisconsin–Madison. After viral infection, the cells were washed twice with fetal bovine serum (100% w/v) and twice with PBS. Then the cells were stained with anti-mouse CD64 APC-eFluor 780 to identify the alveolar macrophages (Figs. 1 and 2). Then, the cells were analyzed by flow cytometry and influenza virus–infected cells were quantified. Similar studies were conducted using the same approach with bone marrow–derived macrophages (BMDMs) (4 × 10^6^ cells) that were generated from WT and CD209d/e^−/−^ mice as described.^18^

### Quantification of influenza virus infected macrophages in the lung

Lung sections were fixed in 10% buffered formalin for 48 hours and then embedded in paraffin. The 5-μm sections were blocked with 5% normal donkey serum (017-000-121, RRID: AB_2337258; Jackson ImmunoResearch Laboratories) in PBS for 30 min at room temperature. Lungs were then incubated overnight at room temperature with goat anti-influenza A virus (AB1074, RRIDAB_90475; Millipore) and rat anti-mouse F4/80 (clone Cl: A3-1, MCA497G, RRID: AB_872005; Bio-Rad). Bound-secondary antibodies were detected with Alexa Fluor 568 donkey anti-goat Ig G (A11057, RRID: AB_2534104; Thermo Fisher Scientific) and Alexa Fluor 647 donkey anti-rat Ig G (712-606-153, RRID: AB_2340696; Jackson ImmunoResearch Laboratories) antibodies for 1 hour at room temperature. Sections were washed twice with PBS and mounted with Vectashield mounting medium with DAPI counterstain (H-1200-10; Vector Laboratories). Representative 200× images were taken with a Zeiss Axioplan Microscope and recorded with a Hamamatsu Camera. F4/80^+^ Influenza A^+^ macrophages were counted in 3 random 200× fields per mouse (C57BL/6 mice: n = 9, 27 fields at 200×, CD209d/e^−/−^ mice: n = 10, 30 fields at 200×) in alveolar areas distal to bronchi and vessels. Given neutrophils express F4/80, small cells with multilobed nuclei and neutrophil like morphology were excluded from the morphometric analysis. Only middle size and large unilobed cells positive for F4/80 and influenza A virus were counted in the alveolar spaces.

### Influenza virus and TLR ligand studies

BMDMs were generated from WT and CD209d/e^−/−^ mice as described previously.^18^ BMDMs were plated at 1 × 10^6^ cells/mL in 1 mL in 24-well tissue culture-treated plates, rested overnight at 37 °C in 5% CO_2_, then treated with influenza A/PR/8/34 virus, Poly I:C (10 µg/mL) TLR3 ligand, or CpG ODN1585 (5 µM/mL) TLR9 ligand.^20^^,^^21^ Twenty-four hours later, cell culture supernatants were harvested, and cells were lysed in RLT buffer and frozen at −80°C for RNA extraction using a RNeasy mini kit (Qiagen). RNA was converted to complementary DNA using reverse transcription super-mix (Bio-Rad Laboratories). Gene expression was analyzed by quantitative RT-PCR using TaqMan probes and primers (Table S1). Cell supernatants were analyzed by Bio-Plex assay (Bio-Rad Laboratories).

### Statistical analysis

All analyses were performed using GraphPad Prism software (GraphPad Software version 10.3.0). All studies were performed a minimum of twice with data combined. Student’s *t* test was used for analysis between 2 means and differences were deemed significant if *P* ≤ 0.05. For more than 2 means, 1-way analysis of variance was used followed by post hoc Tukey test. All figures represent data as mean ± SEM.

## Results

### CD209d/e^−/−^ mice have reduced weight loss and influenza viral burden

Influenza virus activates the immune system through various pattern recognition receptors and induces proinflammatory cytokines and chemokines to control the virus.^7^^,^^22^^,^^23^ In this study, we examined if missing CD209d and CD209e would have an effect on influenza disease severity. We infected WT and CD209d/e^−/−^ mice with 100 pfus of influenza virus in 50 µL of sterile PBS (day 0). Weight loss serves as an indicator of infection severity and morbidity. Therefore, we determined the weight loss after influenza viral infection. By day 7, CD209d/e^−/−^ mice lost significantly less weight when compared with WT mice in response to influenza virus infection (Fig. 1A). CD209d/e^−/−^ lost about 13% of their starting weight while the WT group lost approximately 19% of their starting weight following influenza viral infection (Fig. 1A).

Next, we tested whether the absence of CD209d/e had an effect on the viral burden during influenza viral infection. We measured the RNA expression of influenza viral matrix (M) protein and viral titers by plaque assay from influenza virus-infected WT and CD209d/e^−/−^ mice. We found the expression of M protein and viral titers were significantly decreased in CD209d/e^−/−^ mice when compared with WT mice (Fig. 1B and C). These data suggest that the CD209d/e^−/−^ mice have less viral load that may result in less weight loss when compared with WT mice in response to influenza viral infection.

### CD209d/e^−/−^ macrophages have reduced viral binding and uptake during influenza virus infection

Previous study has shown that DC-SIGN (CD209) and L-SIGN (CD209L) are endocytic receptors for influenza viral entry.^24^ In our study, we found decreased viral burden in CD209d/e^−/−^ mice infected with influenza virus. We next tested whether influenza viral binding was compromised in the absence of CD209d/e on macrophages. To test this, we isolated BAL cells from naïve mice and tested the macrophage binding ability by using the fluorescent reporter Color-flu strain. We found significantly reduced frequency of Color-flu virus–positive CD209d/e^−/−^ macrophages when compared with WT macrophages (Fig. 1D). We further tested the influenza virus binding ability in BMDMs from WT and CD209d/e^−/−^ mice. Similar to the alveolar macrophages, we found significantly decreased frequency of influenza virus–positive BMDMs in CD209d/e^−/−^ compared with WT BMDMs (Fig. 1E). Overall, these data suggest that macrophages lacking CD209d/e have impaired influenza virus uptake associated with reduced infection burden in the lung.

### CD209d/e^−/−^ mice have reduced TLR expression during influenza virus infection

TLR3, TLR7, TLR9, and RIG1 are known to be involved in sensing influenza viral antigens and activating downstream signaling pathways.^5^^,^^25^^,^^26^ To determine whether reduced viral burden and viral sensing in CD209d/e mice had any effect on TLR and RIG1 expression, we tested the expression of *Tlr3*, *Tlr7*, *Tlr9*, and *Rig1* in WT and CD209d/e^−/−^ mice during influenza viral infection. Influenza virus infection significantly increased expression of *Tlr3*, *Tlr7*, *Tlr9*, and *Rig1* in WT and CD209d/e^−/−^ mice compared with uninfected levels (Fig. 2A–D). We found that expression of *Tlr3* and *Tlr9* was significantly decreased in CD209d/e^−/−^ mice when compared with WT mice during influenza viral infection (Fig. 2A and B). We found no differences in the expression of *Tlr7* and *Rig1* between WT and CD209d/e^−/−^ mice (Fig. 2C and D). These data demonstrate that the absence of CD209d/e modulated *Tlr3* and *Tlr9* expression during the influenza viral infection.

To further test the impact of CD209d/e on TLR signaling, we generated BMDMs and treated with influenza A/PR/8/34 virus, Poly I:C (TLR3 agonist), or CpG ODN1585 (TLR9 agonist) for 24 h and the expression of *Tlr3*, *Tlr7*, *Tlr9*, and *Rig1* were analyzed. We found that influenza viral infection increased the expression of *Tlr3*, *Tlr9*, and *Rig1* in BMDMs from both WT and CD209^−/−^ mice regardless of genotype (Fig. 2E–H). We further found that *Tlr3* and *Tlr9* expression was suppressed in response to Poly I:C treatment in BMDMs from CD209d/e^−/−^ mice when compared with BMDMs from WT mice (Fig. 2E and F). We also found that *Tlr9* and *Rig1* expression was suppressed in CpG ODN1585-treated BMDMs from CD209d/e^−/−^ mice when compared with BMDMs from WT mice (Fig. 2F and H). However, we found no differences in *Tlr7* in response to influenza virus, Poly I:C, or CpG ODN1585 treatment in WT or CD209d/e^−/−^ BMDMs (Fig. 2G). These data suggest that CD209d/e modulates TLR3, TLR9, and RIG1 signaling pathways in macrophages.

### Expression of interferon response genes are decreased in CD20d/e^−/−^ mice during influenza virus infection

**Figure 3.**
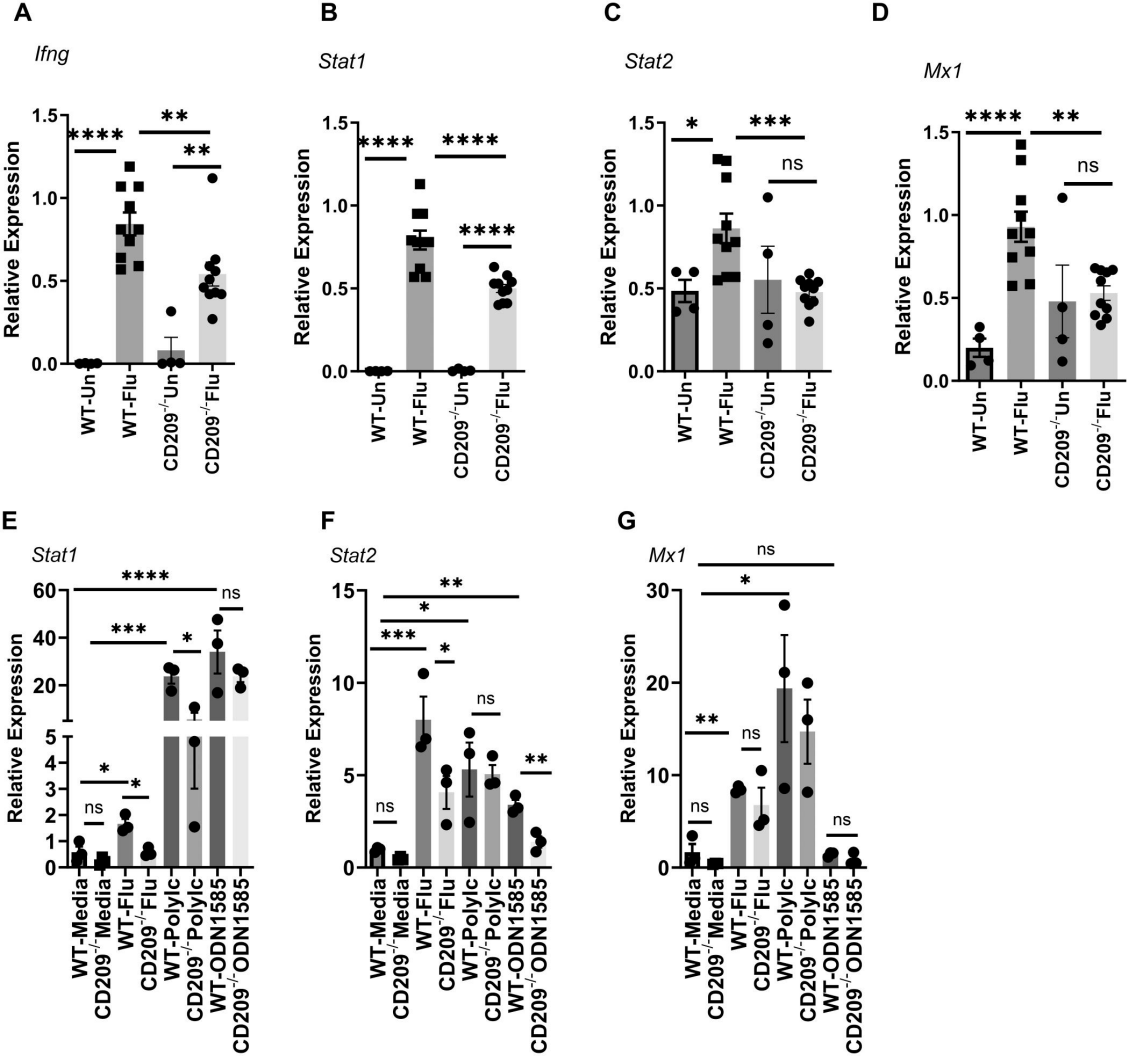
Expression of IFN genes were decreased in CD209d/e^−/−^ mice during influenza virus infection. WT and CD209d/e^−/−^ (CD209^−/−^) mice were infected with 10^2^ PFUs of influenza A PR/8/34 virus (Flu) for 7 d. (A–D) Expression of *Ifnγ*, *Stat1*, *Stat2*, and *Mx1* in the lung was measured from uninfected (Un) and infected mice by RT-PCR. (E–G) BMDMs from WT and CD209d/e^−/−^ mice were treated with influenza A/PR/8/34 virus, Poly I:C (10 µg/mL), or CpG ODN1585 (5 µM/mL) for 24 h, and *Stat1*, *Stat2*, and *Mx1*expression levels were measured by RT-PCR. Data are represented as mean ± SEM. Significance was tested by analysis of variance and Tukey test. Each experiment was independently performed 2 or more times, and combined data are shown. *P < 0.05, **P < 0.01, ***P < 0.001, ****P < 0.0001. ns, not significant.

In response to influenza viral infection, the host immune system induces type I, II and III interferons (IFNs) and a variety of proinflammatory cytokines and chemokines. These IFNs inhibit viral replication by inducing IFN-stimulated genes (ISGs), such as MX dynamin like GT Pase1 (Mx1), via JAK-dependent phosphorylation of Stat1 and Stat2.^27^ To determine whether the absence of CD209d/e had an effect on IFN responses, we measured the expression of *Ifnβ*, *Ifnλ*, *Ifnγ*, *Stat1*, *Stat2*, and *Mx1* on day 7 following influenza virus infection. Influenza virus significantly induced expression of *IFNγ*, *Stat1*, *Stat2*, and *Mx1* in WT mice and *IFNγ* and *Stat1* in CD209d/e^−/−^ mice compared with uninfected control mice (Fig. 3A–D). We found significantly decreased expression of *IFNγ*, *Stat1*, *Stat2*, and *Mx1* in CD209d/e^−/−^ mice when compared with WT mice during influenza viral infection (Fig. 3A–D). No differences were observed in expression of *IFNβ* and *IFNλ* in between influenza virus infected WT and CD209d/e^−/−^ mice (Fig. S3). These data suggest that the IFN pathway gene expression was decreased in the absence of CD209d/e during influenza viral infection.

To further analyze the role CD209d/e on IFN responses, we analyzed the expression of *Stat1*, *Stat2*, and *Mx1* in WT and CD209d/e^−/−^ BMDMs treated with influenza virus, Poly I:C, or CpG ODN1585. We found the influenza viral infection or TLR ligand increased the expression of *Stat1*, *Stat2*, and *Mx1* in BMDMs from WT and CD209d/e^−/−^ mice (Fig. 3E–G). Influenza virus induced expression of *Stat1* and *Stat2* was significantly decreased in BMDMs from CD209d/e^−/−^ mice when compared with WT mice (Fig. 3E and F). Poly I:C–induced *Stat1* expression and CpG ODN1585–induced *Stat2* expression were suppressed in BMDMs from CD209d/e^−/−^ when compared with BMDMs from WT mice (Fig. 3E and F). These data demonstrate that the CD209d/e modulates genes involved in IFN signaling pathways during influenza viral infection.

### Lung inflammatory immune responses are reduced in CD209d/e^−/−^ mice during influenza virus infection

**Figure 4.**
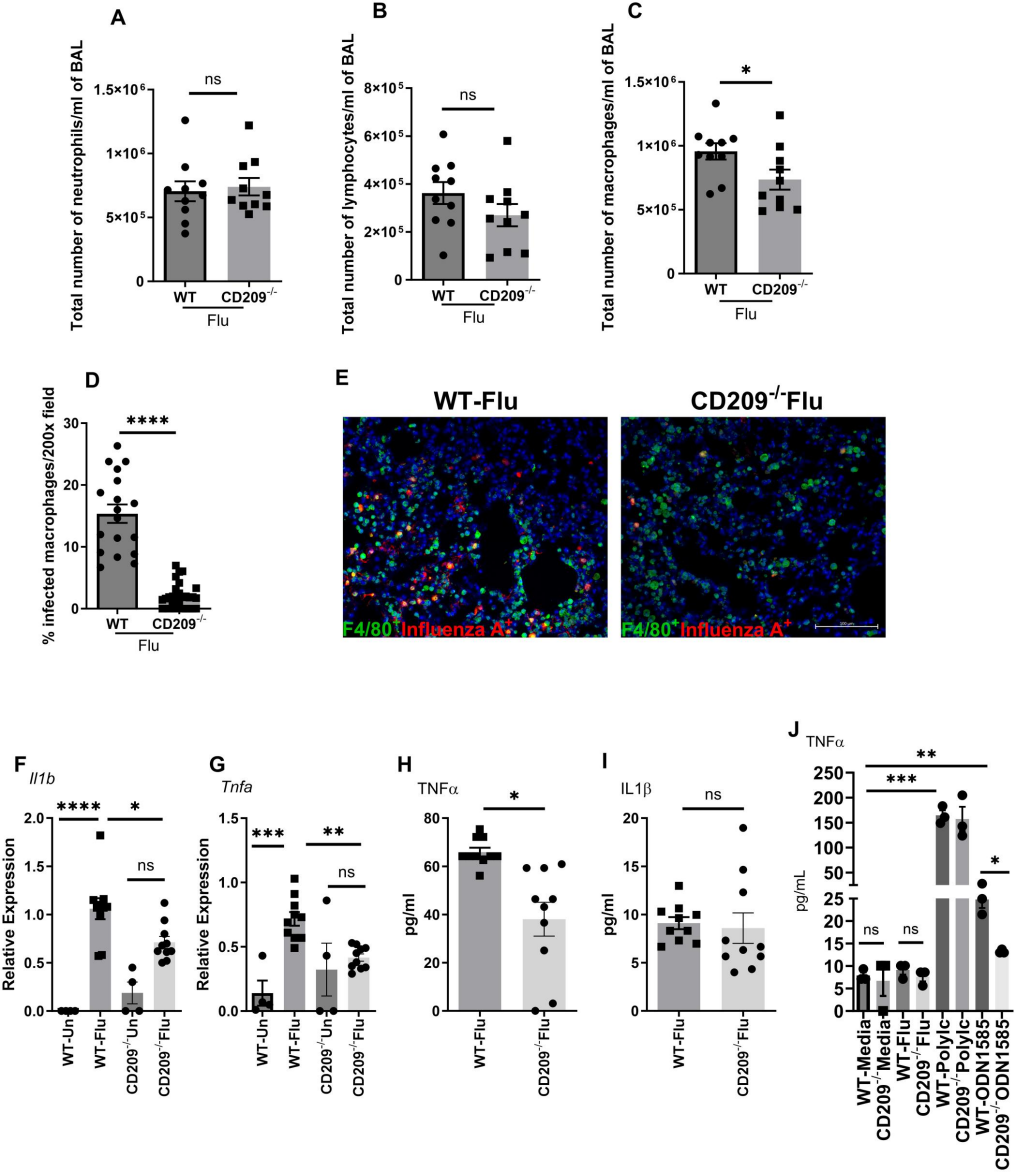
Lung inflammatory immune response is decreased CD209d/e^−/−^ mice during influenza virus infection. WT and CD209d/e^−/−^ (CD209^−/−^) mice were infected with 10^2^ PFUs of influenza A PR/8/34 virus (Flu) for 7 d. BAL cells were stained and differential cells were counted as described in the Materials and Methods. (A) Total number of neutrophils, (B) lymphocytes, and (C) macrophages were measured in BAL. (D, E) Paraffin-fixed lung sections were stained and F4/80^+^ influenza A^+^ macrophages were analyzed by immunofluorescence (200× magnification). (F, G) Expression of *Il1β* and *Tnfα* in the lung were measured from uninfected (Un) and influenza virus–infected mice by RT-PCR. (H, I) Levels of TNFα and interleukin (IL)-1β from influenza virus–infected mice were measured by Bio-Plex assay. (J) BMDMs from WT and CD209d/e^−/−^ mice were treated with influenza A/PR/8/34 virus, Poly I:C (10 µg/mL), or CpG ODN1585 (5 µM/mL) for 24 h, and TNFα expression was measured by Bio-Plex assay. Data are represented as mean ± SEM. Significance was tested by unpaired *t* test or analysis of variance and Tukey test. Each experiment was independently performed 2 or more times, and combined data are shown. *P < 0.05, **P < 0.01, ***P < 0.001, ****P < 0.0001. ns, not significant.

In response to influenza viral entry, epithelial and immune cells induce pro-inflammatory cytokines and chemokines such as interleukin-1β, interleukin-6, tumor necrosis factor α (TNFα), CCL2, CCL3, and CCL5, which recruit macrophages and neutrophils to the site of infection to control the virus.^5^ The inflammatory response may become uncontrollable, culminating in a cytokine storm that can cause severe illness. In our study, we examined whether CD209d/e had an effect on the lung inflammatory immune response. We first measured the differential cell counts from BAL to measure airway inflammation within the lung. We found no significant differences in total neutrophils and lymphocytes in BAL fluid between WT and CD209d/e^−/−^ mice (Fig. 4A and B). However, CD209d/e^−/−^ mice had significantly reduced macrophage recruitment to the lungs (Fig. 4C). We confirmed this significant decrease in macrophages by examining influenza virus-positive macrophages in CD209d/e^−/−^ mice when compared with WT mice (Fig. 4D and E). These data suggest that the CD209d/e^−/−^ mice recruit a smaller number of macrophages during influenza viral infection.

Next, we measured whether the lower viral burden and lesser macrophage recruitment translated into less induction of proinflammatory cytokines in lung tissue. We found that the expression of *Tnfα* and *Il1β*, and protein levels of TNFα were significantly decreased in CD209d/e^−/−^ mice when compared with WT mice during influenza viral infection (Fig. 4F–I). These data suggest that the lack of CD209d/e modulated the proinflammatory immune response to influenza viral infection. We further analyzed the effect of CD209d/e on cytokine regulation in macrophages during influenza viral infection or TLR stimulation in BMDMs. We found decreased production of TNFα in response to CpG ODN1585 in CD209d/e^−/−^ BMDMs when compared with WT BMDMs (Fig. 4J). These data suggest that CD209d/e regulates proinflammatory cytokine regulation through the TLR9 signaling pathway.

**Figure 5.**
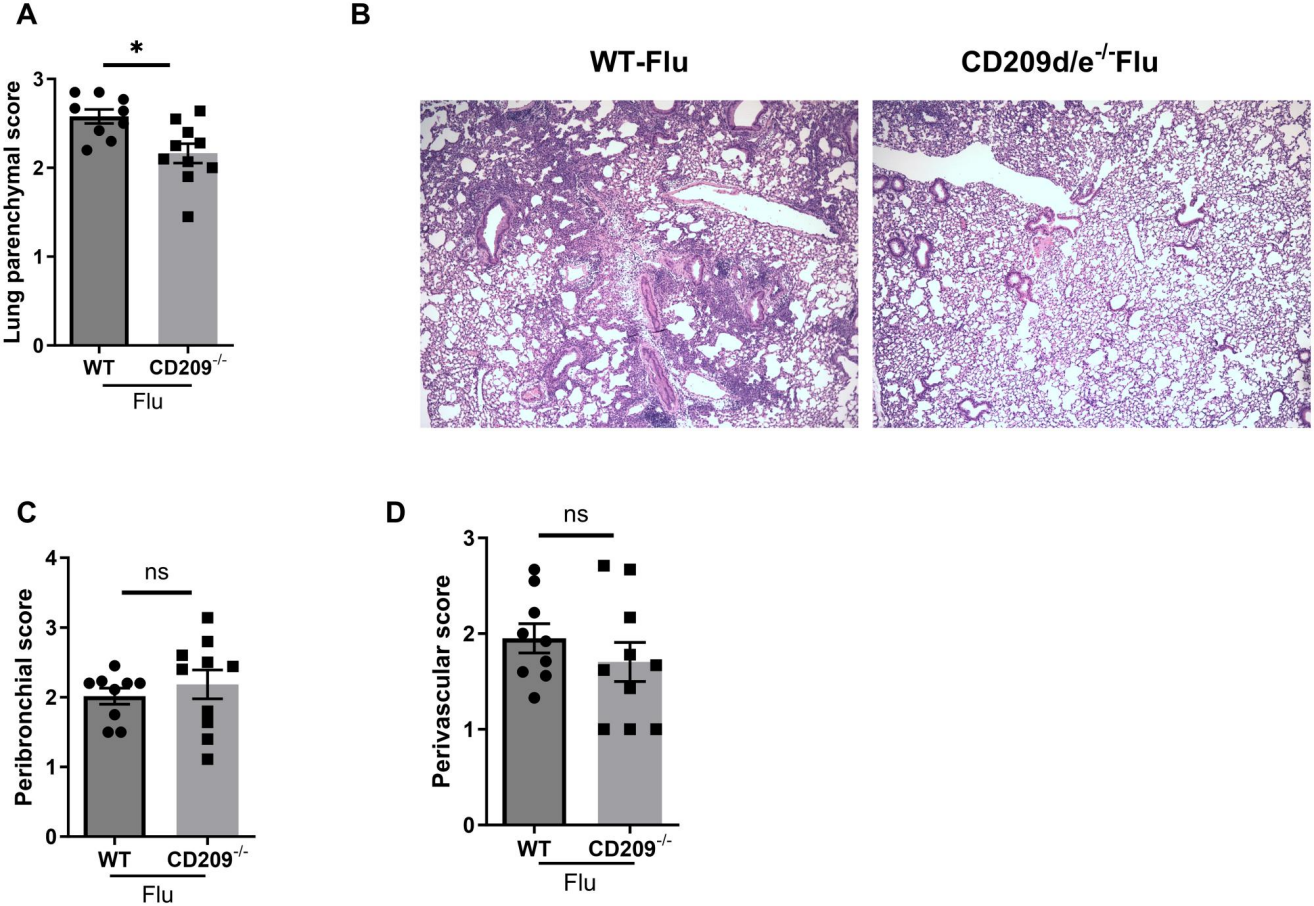
Lung parenchymal inflammation is decreased in CD209d/e^−/−^ mice during influenza virus infection. WT and CD209d/e^−/−^ (CD209^−/−^) mice were infected with 10^2^ PFUs of influenza A PR/8/34 virus (Flu) for 7 d. (A) Lung parenchymal, (C) peribronchial, and (D) perivascular inflammation scores were measured. (B) Representative lung histology, 40× magnification. Data are represented as mean ± SEM. Significance was tested by unpaired *t* test. Each experiment was independently performed 2 or more times, and combined data are shown. *P < 0.05. ns, not significant.

Finally, we determined whether the decreased viral burden, number of macrophages, and inflammatory cytokines in CD209d/e^−/−^ mice had an effect on lung pathology during influenza viral infection. We found that the lung parenchymal inflammation was significantly decreased in CD209d/e^−/−^ mice when compared with WT mice (Fig. 5A and B). No significant differences were observed in peribronchiolar and perivascular inflammation scoring between WT and CD209d/e^−/−^ mice (Fig. 5C and D). These data show that the absence of CD209d/e reduced inflammatory immune responses during influenza viral infection.

## Discussion

Influenza viral infection induces interferons and a variety of inflammatory cytokines and chemokines in the host.^5^ These chemokines recruit macrophages and neutrophils to control the virus.^28^ However, excessive inflammation can lead to cytokine storm and acute respiratory distress syndrome. Here, we tested whether the C-type lectin membrane proteins CD209d/e had an effect on influenza viral infection. It has been shown previously that the CD209 is an endocytic receptor for viral infection.^24^^,^^29^ In our study, we found that weight loss and the expression of viral M protein were decreased in CD209d/e^−/−^ mice when compared with WT mice. Next, we measured viral binding to lung and BMDMs by using Color-flu. We found decreased viral binding in CD209d/e^−/−^ macrophages when compared with WT macrophages. This was confirmed by in vivo staining of influenza virus–infected macrophages. These data suggest that the CD209d/e is involved in viral sensing and/or binding during influenza entry. It has been shown that DC-SIGN and L-SIGN are able to restore the influenza viral binding ability of sialic acid–defective Lec2 Chinese hamster ovary cells.^24^^,^^30^ Further, it has been shown that the DC-SIGN mediated binding is dependent on the extent of glycosylation of viral hemagglutinin.^31^ Similarly, studies have shown that CD209a on dendritic cells can detect influenza virus and drive protective immune responses in mice.^32^^,^^33^ CD209a has been shown to be required for upper airway activation of natural killer cells by influenza virus, and lacking CD209a resulted in increased viral replication. Our results concerning CD209d/e deletion resulted in decreased viral burden, which is inconsistent with these findings regarding the related CD209a protein and suggests a distinct functional niche.

TLR3, TLR7, TLR9, and RIG1 are known to be involved in sensing influenza viral antigens and activating downstream signaling pathways.^5^^,^^25^^,^^34^ In our study, we found the expression of *Tlr3* and *Tlr9* were significantly decreased in influenza virus infected CD209d/e^−/−^ mice when compared with WT mice. No differences were observed in the expression of *Tlr7* and *Rig1* between WT and CD209d/e^−/−^ mice. Using BMDMs, we found decreased expression of *Tlr3* and *Tlr9* in Poly I:C–treated CD209d/e^−/−^ BMDMs when compared with WT BMDMs. Similarly, we found decreased expression of *Tlr9* in ODN1585-treated CD209d/e^−/−^ BMDMs when compared with WT BMDMs. These data suggest that in the absence of CD209d/e, a decrease in viral binding/sensing may affect pathogen-associated molecular pattern sensing by TLR3 and TLR9 during influenza virus infection. Next, we examined the interferon response to influenza viral infection in WT and CD209d/e^−/−^ mice. Type I IFNs (IFNα/β) bind to IFNaR1 and IFNaR2 receptors, leading to phosphorylation of STAT1 and STAT2, and transcription of ISGs. Type II IFN (IFNγ) binds to IFNγR1 and IFNγR2 and activates only STAT1 signaling.^35^^,^^36^ Type III IFNs signal through IFNLR and IL10R2 to induce ISGs similar to those induced by type I IFN.^36^ In our study, we found significantly decreased expression of *Ifnγ*, *Stat1*, *Stat2*, and *Mx1* in influenza virus–infected CD209d/e^−/−^ mice when compared with WT mice. However, we found no differences in the expression of *Ifnβ* and *Ifnλ* in between WT and CD209d/e^−/−^ mice. Using BMDMs from WT and CD209d/e^−/−^ mice, we showed that Poly I:C–induced *Stat1* and CpG ODN1585–induced *Stat2* expression was impacted by deletion of CD209d/e. Influenza virus–induced *Stat1* and *Stat2* expression in BMDMs was decreased in CD209d/e^−/−^ mice compared with WT mice. These data suggest that IFN signaling pathways are suppressed in CD209d/e^−/−^ when compared with WT mice. This may be due to decreased viral sensing or burden in CD209d/e^−/−^ compared with WT macrophages and mice. Our findings suggest that the CD209d/e interacts with type II IFN and its associated signaling during influenza viral infection. However, the decrease in *Stat2* expression observed in CD209d/e^−/−^ mice suggests that there are possible downstream signaling interactions between Stat2 and CD209d/e during influenza viral infection.

Finally, we determined whether CD209d/e had an effect on lung inflammation. We found a decreased number of BAL macrophages in CD209d/e^−/−^ mice when compared with WT mice. These data suggest that decreased macrophages may be due to decreased viral infection in CD209d/e^−/−^ mice. We further confirmed a lower number of influenza virus–positive macrophages in the lung in CD209d/e^−/−^ mice when compared with WT mice. We also found decreased expression of proinflammatory cytokines such as *Tnfα* and *Il1β* in CD209d/e^−/−^ mice when compared with WT mice. Previously it has been shown that dendritic cells use CD209a to detect influenza virus and induce chemokines such as CCL5, CXCL9, and CXCL10.^32^^,^^33^ In our studies, we found less parenchymal lung inflammation in CD209d/e^−/−^ mice when compared with WT mice. These data suggest that the decreased viral replication in the absence of CD209d/e resulted in less lung inflammation. Overall, our data suggest that the CD209d/e is involved in viral binding by macrophages, which in turn increases sensing through TLRs, type II IFN induction, lung inflammation, and lung injury during influenza viral infection.

## Supplementary Material

vlae001_Supplementary_Data

## Data Availability

The raw datasets generated and/or analyzed during the current study are available from the corresponding author upon request.

## References

[1] Molinari NA , et al The annual impact of seasonal influenza in the US: measuring disease burden and costs. Vaccine. 2007;25:5086–5096. 17544181 10.1016/j.vaccine.2007.03.046

[2] Van Kerkhove MD , Hirve S, Koukounari A, Mounts AW; H1N1pdm Serology Working Group. Estimating age-specific cumulative incidence for the 2009 influenza pandemic: a meta-analysis of A(H1N1)pdm09 serological studies from 19 countries. Influenza Other Respir Viruses. 2013;7:872–886. 23331969 10.1111/irv.12074 PMC5781221

[3] Heron M. Deaths: leading causes for 2017. Natl Center Health Stat. 2019;68:1–77. 32501203

[4] Ballinger MN , Standiford TJ. Postinfluenza bacterial pneumonia: host defenses gone awry. J Interferon Cytokine Res. 2010;30:643–652. 20726789 10.1089/jir.2010.0049 PMC4367524

[5] Iwasaki A , Pillai PS. Innate immunity to influenza virus infection. Nat Rev Immunol. 2014;14:315–328. 24762827 10.1038/nri3665 PMC4104278

[6] Mifsud EJ , Kuba M, Barr IG. Innate immune responses to influenza virus infections in the upper respiratory tract. Viruses. 2021;13:2090. 34696520 10.3390/v13102090 PMC8541359

[7] Ortiz M , et al The evolutionary history of the CD209 (DC-SIGN) family in humans and non-human primates. Genes Immunity. 2008;9:483–492. 18528403 10.1038/gene.2008.40 PMC2701223

[8] Garcia-Vallejo JJ , van Kooyk Y. The physiological role of DC-SIGN: a tale of mice and men. Trends Immunol. 2013;34:482–486. 23608151 10.1016/j.it.2013.03.001

[9] Powlesland AS , et al Widely divergent biochemical properties of the complete set of mouse DC-SIGN-related proteins. J Biol Chem. 2006;281:20440–20449. 16682406 10.1074/jbc.M601925200

[10] Gopal R , et al STAT2 signaling regulates macrophage phenotype during influenza and bacterial super-infection. Front Immunol. 2018;9:2151. 30337919 10.3389/fimmu.2018.02151 PMC6178135

[11] Robinson KM , et al Influenza A exacerbates Staphylococcus aureus pneumonia by attenuating IL-1beta production in mice. J Immunol. 2013;191:5153–5159. 24089191 10.4049/jimmunol.1301237 PMC3827735

[12] McHugh KJ , Mandalapu S, Kolls JK, Ross TM, Alcorn JF. A novel outbred mouse model of 2009 pandemic influenza and bacterial co-infection severity. PloS One. 2013;8:e82865. 24324838 10.1371/journal.pone.0082865 PMC3855784

[13] McHugh KJ , Mandalapu S, Kolls JK, Ross TM, Alcorn JF. A novel outbred mouse model of 2009 pandemic influenza and bacterial co-infection severity. PLoS One. 2013;8:e82865. 24324838 10.1371/journal.pone.0082865 PMC3855784

[14] Karakus U , Crameri M, Lanz C, Yanguez E. Propagation and titration of influenza viruses. Methods Mol Biol. 2018;1836:59–88. 30151569 10.1007/978-1-4939-8678-1_4

[15] Rao X , Huang X, Zhou Z, Lin X. An improvement of the 2ˆ(-delta delta CT) method for quantitative real-time polymerase chain reaction data analysis. Biostat Bioinforma Biomath. 2013;3:71–85. 25558171 PMC4280562

[16] Manni ML et al Bromodomain and extra-terminal protein inhibition attenuates neutrophil-dominant allergic airway disease. Sci Rep. 2017;7:43139. 28233801 10.1038/srep43139 PMC5324049

[17] Cipolla EM et al Heterotypic influenza infections mitigate susceptibility to secondary bacterial infection. J Immunol. 2022;209:760–771. 35914833 10.4049/jimmunol.2200261 PMC9378502

[18] Constantinesco NJ et al Sodium-glucose cotransporter-2 inhibitor, empagliflozin, suppresses the inflammatory immune response to influenza infection. Immunohorizons. 2023;7:861–871. 38112660 10.4049/immunohorizons.2300077 PMC10759161

[19] Robinson KM et al Influenza A virus exacerbates Staphylococcus aureus pneumonia in mice by attenuating antimicrobial peptide production. J Infect Dis. 2014;209:865–875. 24072844 10.1093/infdis/jit527 PMC3935471

[20] Anker SD , Butler J, Packer M. Empagliflozin in heart failure with a preserved ejection fraction. Reply. N Engl J Med. 2022;386:e57. 10.1056/NEJMc2118470 35613034

[21] Jiang T et al Activation of TLR9 signaling suppresses the immunomodulating functions of CD55(lo) fibroblastic reticular cells during bacterial peritonitis. Front Immunol. 2024;15:1337384. 38827745 10.3389/fimmu.2024.1337384 PMC11140099

[22] Serrano-Gomez D et al Dendritic cell-specific intercellular adhesion molecule 3-grabbing nonintegrin mediates binding and internalization of Aspergillus fumigatus conidia by dendritic cells and macrophages. J Immunol. 2004;173:5635–5643. 15494514 10.4049/jimmunol.173.9.5635

[23] Boehme KW , Compton T. Innate sensing of viruses by toll-like receptors. J Virol. 2004;78:7867–7873. 15254159 10.1128/JVI.78.15.7867-7873.2004 PMC446107

[24] Gillespie L et al Endocytic function is critical for influenza A virus infection via DC-SIGN and L-SIGN. Sci Rep. 2016;6:19428., 26763587 10.1038/srep19428 PMC4725901

[25] Wu S , Metcalf JP, Wu W. Innate immune response to influenza virus. Curr Opin Infect Dis. 2011;24:235–240. 21330918 10.1097/QCO.0b013e328344c0e3

[26] Han Y , Bo ZJ, Xu MY, Sun N, Liu DH. The protective role of TLR3 and TLR9 ligands in human pharyngeal epithelial cells infected with influenza A virus. Kor J Physiol Pharmacol. 2014;18:225–231. 10.4196/kjpp.2014.18.3.225 PMC4071175 24976762

[27] Lee AJ , Ashkar AA. The dual nature of type I and Type II interferons. Front Immunol. 2018;9:2061. 30254639 10.3389/fimmu.2018.02061 PMC6141705

[28] Ichinohe T , Lee HK, Ogura Y, Flavell R, Iwasaki A. Inflammasome recognition of influenza virus is essential for adaptive immune responses. J Exp Med. 2009;206:79–87. 19139171 10.1084/jem.20081667 PMC2626661

[29] Yu L et al High doses of recombinant mannan-binding lectin inhibit the binding of influenza A(H1N1)pdm09 virus with cells expressing DC-SIGN. Apmis. 2017;125:655–664. 28493491 10.1111/apm.12695

[30] Londrigan SL et al N-linked glycosylation facilitates sialic acid-independent attachment and entry of influenza A viruses into cells expressing DC-SIGN or L-SIGN. J Virol. 2011;85:2990–3000. 21191006 10.1128/JVI.01705-10 PMC3067946

[31] Hillaire MLB et al Binding of DC-SIGN to the hemagglutinin of influenza A viruses supports virus replication in DC-SIGN expressing cells. PLos ONE. 2013;8:e56164., 23424649 10.1371/journal.pone.0056164 PMC3570528

[32] Gonzalez SF et al Capture of influenza by medullary dendritic cells via SIGN-R1 is essential for humoral immunity in draining lymph nodes. Nat Immunol. 2010;11:427–434. 20305659 10.1038/ni.1856 PMC3424101

[33] Palomino-Segura M et al Protection against influenza infection requires early recognition by inflammatory dendritic cells through C-type lectin receptor SIGN-R1. Nat Microbiol. 2019;4:1930–1940. 31358982 10.1038/s41564-019-0506-6 PMC6817362

[34] Essayas H et al Toll-like receptor-9 activation promotes persistent inflammation in the lung during influenza infection. Am J Respir Crit Care Med. 2023;207:A5608.

[35] Platanias LC. Mechanisms of type-I- and type-II-interferon-mediated signalling. Nat Rev Immunol. 2005;5:375–386. 15864272 10.1038/nri1604

[36] Durbin JE et al Type I IFN modulates innate and specific antiviral immunity. J Immunol. 2000;164:4220–4228. 10754318 10.4049/jimmunol.164.8.4220

