# Supplementary material for: CD209d/e promotes inflammation and lung injury during influenza virus infection
Source: Immunohorizons. 2025 Jan 23;9(1):vlae001. doi: 10.1093/immhor/vlae001 (PMC11841971; doi:10.1093/immhor/vlae001)
Supplement: vlae001_Supplementary_Data [file vlae001_supplementary_data.zip › 40be5_IMMHOR-24-00034-s01.pdf]

Supplementary Figure-1

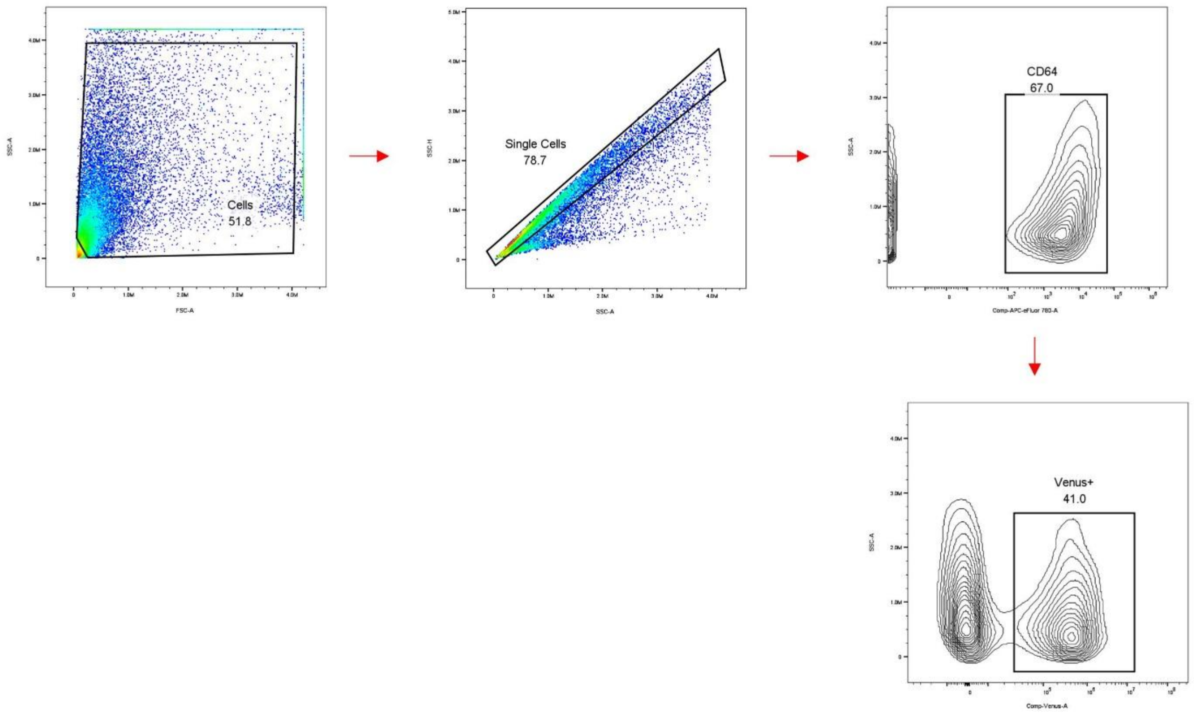

Supplementary Figure 1. Flow cytometry analysis of influenza viral binding to macrophages.

BAL cells were isolated from WT and CD209d/e$^{-/-}$ mice, infected with Color Flu (Venus, MOI of 1). Single cells were initially gated, and further gated on CD64^{+} cells, and Venus^{+} cells using Flow Jo Software. Representative flow cytometry plots are shown.

Supplementary Figure-2

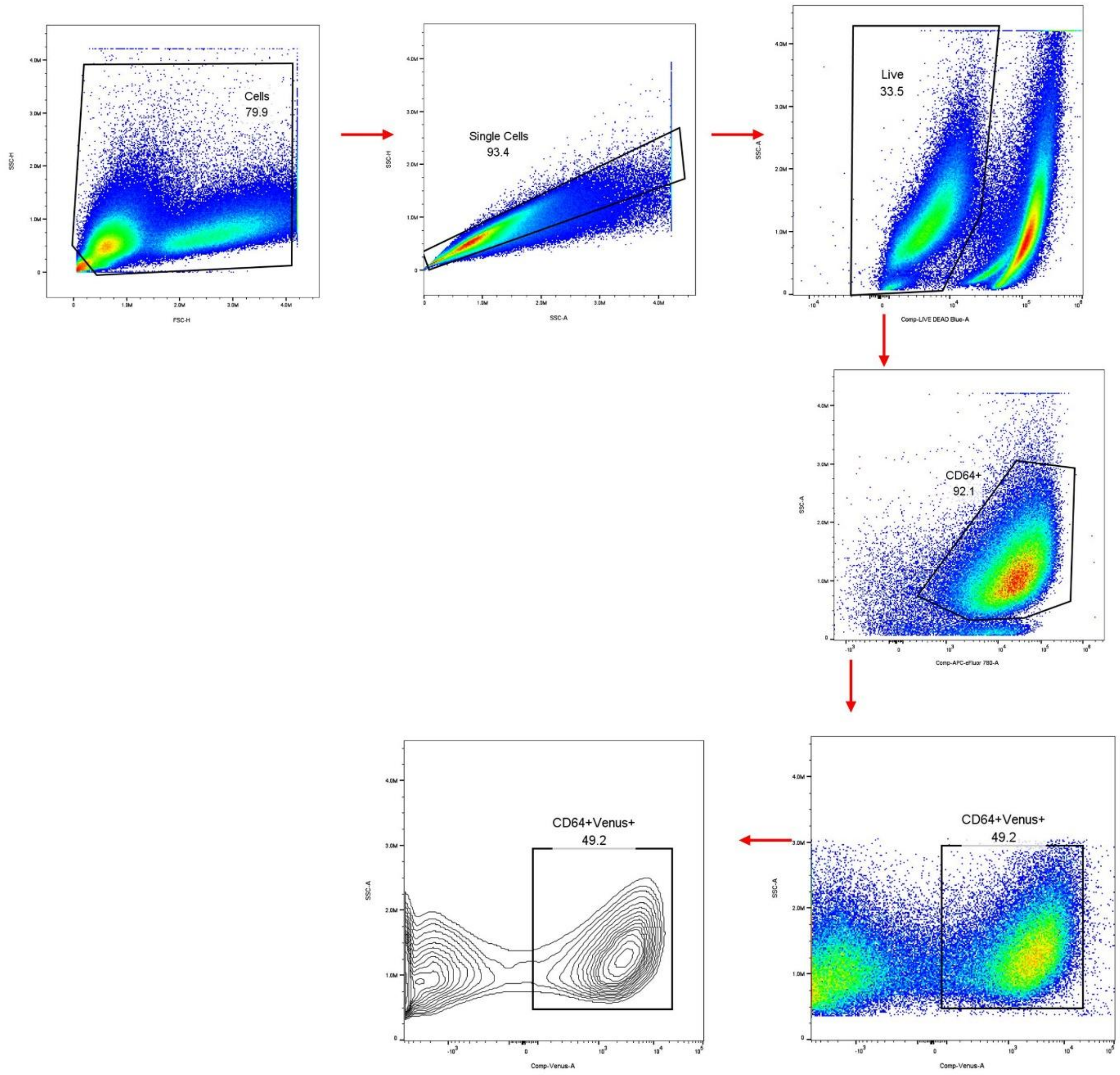

Supplementary Figure 2. Flow cytometry analysis of influenza viral binding to BMDMs.

BMDMs were isolated from WT and CD209d/e$^{-/-}$ mice, infected with Color Flu (Venus, MOI of 1). Single cells were initially gated, and further gated on CD64^{+} cells, and Venus^{+} cells using Flow Jo Software. Representative flow cytometry plots are shown.

Supplementary Figure-3

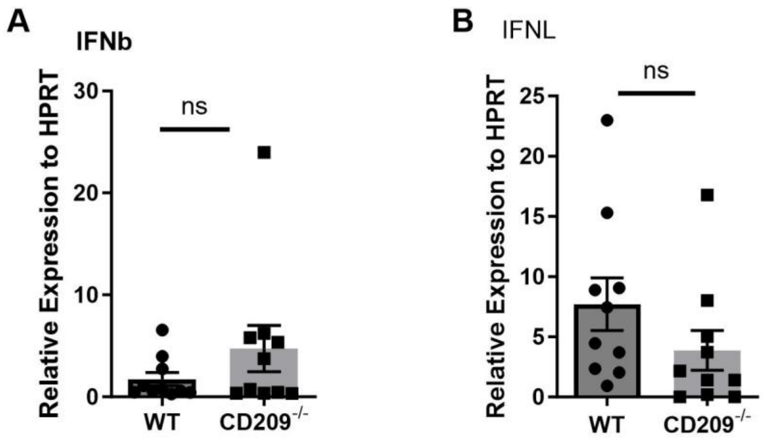

Supplementary Figure 3. Type I and type III IFN responses were not different between WT and CD209d/e$^{-/-}$ mice during influenza virus infection.

WT and CD209d/e$^{-/-}$ mice were infected with 10^2 PFU of influenza A PR/8/34 virus. (A-B) Expression levels of IFNb and IFNL were measured in lung by RT-PCR. Data are represented as mean ± SEM. Significance was tested by unpaired t-test. Each experiment was independently performed two or more times, and combined data are shown. ns = not significant

Supplementary Table 1

No.	Gene	Taqman assay ID
1.	TLR3	Mm01207404_m1
2.	TLR9	Mm07299609_m1
3.	TLR7	Mm04933178_g1
4.	DDX58	Mm01216853_m1
5.	IFNγ	Mm01168134_m1
6.	Stat1	Mm01257286_m1
7.	Stat2	Mm00490880_m1
8.	Mx1	Mm00487796_m1
9.	IL1β	Mm0434288_m1
10.	TNFα	Mm00443288_m1
11.	IFNβ1	Mm00439552
12.	IFNλ3	Mm00663660_g1

Supplementary Table 1: Taqman assays used for RT-PCR analysis.

Supplementary Material 1

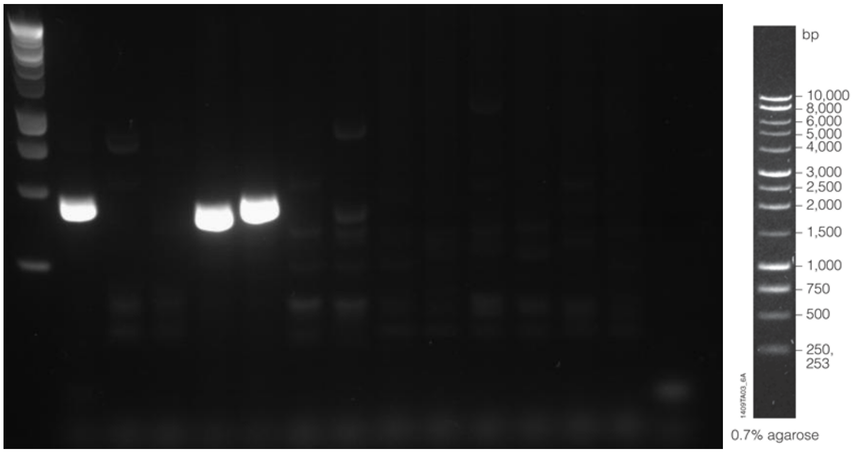

Agarose gel electrophoresis of PCR results in F0 mice. Lane 1: 1kb DNA Ladder (Promega), Lane 2-14: PCR mice 1-13, Lane 15: No DNA control. 2 % agarose gel in 1X TAE. Sequencing results (see below) the fragments are of 456, 434 and 468 bp for mouse #1, 4 and 5, respectively.

Forward and reverse sequences for each mouse were aligned and the contigs were aligned against the mouse genomic reference sequence (UCSC Genome Browser on Mouse Dec. 2011 (GRCm38/mm10) Assembly). Alignment of mouse #1 contig to the mouse chromosome 8 containing the Cd209e and Cd209d genes and confirming deletion of both genes.

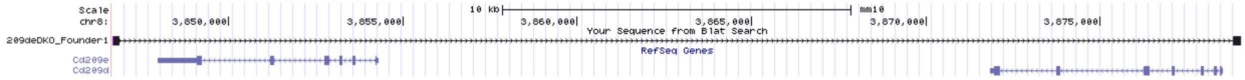

Mouse #1 carries a deletion on chromosome 8 between position 3,846,858 and 3,878,796 (GRCm38/mm10 assembly).

Alignment of CD209d/e$^{-/-}$_Founder1 and chr8:3846668-3879035

---

cDNA CD209deDKO_Founder1

```
cATGGGCTCT GATTCTCAGT CTTCCTGCCT CTTTCATATA GGGATCTCCC   50
TGGCTTAAGT TCATTTATAG ACCTTGTCTC TTGCAGGGTT AAGCACTTAT   100
TCTTTTCACT GTGTGTTCTT TTCACTGTTT TCTTCCATTG AGTTCCTATG   150
GAGAGGCTAG TAGTCTCCCT ACTGGGAGGG AAGTGGCCAC GTGTGCTAAG   200
GATTGGAGAG CCATAACCCA TATTCCTGGT TATTCTGCCC CCACTAACTC   250
ATGCGTCTCT GCTTTGCTAG CATAGGGGCT CGCTGTGATT TCTGCTGACT   300
CTGGTGTATT GACGGACAAA ACATACTCCT ATTAATGCAA TGCCTGCTGC   350
AGATCTACTC ATTTTACCTC TCTATGCTGT AGCACATGAT CATCTTTCCA   400
TTTCTGTACA CCCAGATTAG GTAACAGGCT CA
```

Side by Side Alignment

```
0000002 atgggctctgattctcagtcttcctgcctctttcatatagggatctccct 0000051
>>>>>>> |||||||||||||||||||||||||||||||||||||||||||||||||| >>>>>>>
3846668 atgggctctgattctcagtcttcctgcctctttcatatagggatctccct 3846717

0000052 ggcttaagttcatttatagaccttgtctcttgcagggttaagcacttatt 0000101
>>>>>>> |||||||||||||||||||||||||||||||||||||||||||||||||| >>>>>>>
3846718 ggcttaagttcatttatagaccttgtctcttgcagggttaagcacttatt 3846767

0000102 cttttcactgtgtgttcttttcactgttttcttccattgagttcctatgg 0000151
>>>>>>> |||||||||||||||||||||||||||||||||||||||||||||||||| >>>>>>>
3846768 cttttcactgtgtgttcttttcactgttttcttccattgagttcctatgg 3846817

0000152 agaggctagtagtctccctactgggagggaagtggccacgt 0000192
>>>>>>> ||||||||||||||||||||||||||||||||||||||||| >>>>>>>
3846818 agaggctagtagtctccctactgggagggaagtggccacgt 3846858
```

```
0000193 gtgctaaggattggagagccataacccatattcctggttattctgccccc 0000242
>>>>>>> |||||||||||||||||||||||||||||||||||||||||||||||||| >>>>>>>
3878796 gtgctaaggattggagagccataacccatattcctggttattctgccccc 3878845

0000243 actaactcatgcgtctctgctttgctagcataggggctcgctgtgatttc 0000292
>>>>>>> |||||||||||||||||||||||||||||||||||||||||||||||||| >>>>>>>
3878846 actaactcatgcgtctctgctttgctagcataggggctcgctgtgatttc 3878895

0000293 tgctgactctggtgtattgacggacaaaacatactcctattaatgcaatg 0000342
>>>>>>> |||||||||||||||||||||||||||||||||||||||||||||||||| >>>>>>>
3878896 tgctgactctggtgtattgacggacaaaacatactcctattaatgcaatg 3878945

0000343 cctgctgcagatctactcattttacctctctatgctgtagcacatgatca 0000392
>>>>>>> |||||||||||||||||||||||||||||||||||||||||||||||||| >>>>>>>
3878946 cctgctgcagatctactcattttacctctctatgctgtagcacatgatca 3878995

0000393 tctttccatttctgtacacccagattaggtaacaggctca 0000432
>>>>>>> |||||||||||||||||||||||||||||||||||||||| >>>>>>>
3878996 tctttccatttctgtacacccagattaggtaacaggctca 3879035
```
